# Guiding Glucose Management Discussions Among Adults With Type 2 Diabetes in General Practice: Development and Pretesting of a Clinical Decision Support Tool Prototype Embedded in an Electronic Medical Record

**DOI:** 10.2196/17785

**Published:** 2020-09-02

**Authors:** Breanne E Kunstler, John Furler, Elizabeth Holmes-Truscott, Hamish McLachlan, Douglas Boyle, Sean Lo, Jane Speight, David O'Neal, Ralph Audehm, Gary Kilov, Jo-Anne Manski-Nankervis

**Affiliations:** 1 Department of General Practice University of Melbourne Melbourne, Victoria Australia; 2 School of Psychology Deakin University Geelong, Victoria Australia; 3 Australian Centre for Behavioural Research in Diabetes Diabetes Victoria Melbourne Australia; 4 Department of Medicine St Vincent's Hospital University of Melbourne Melbourne Australia

**Keywords:** type 2 diabetes, shared decision making, clinical decision support, general practice, primary care

## Abstract

**Background:**

Managing type 2 diabetes (T2D) requires progressive lifestyle changes and, sometimes, pharmacological treatment intensification. General practitioners (GPs) are integral to this process but can find pharmacological treatment intensification challenging because of the complexity of continually emerging treatment options.

**Objective:**

This study aimed to use a co-design method to develop and pretest a clinical decision support (CDS) tool prototype (GlycASSIST) embedded within an electronic medical record, which uses evidence-based guidelines to provide GPs and people with T2D with recommendations for setting glycated hemoglobin (HbA1c) targets and intensifying treatment together in real time in consultations.

**Methods:**

The literature on T2D-related CDS tools informed the initial GlycASSIST design. A two-part co-design method was then used. Initial feedback was sought via interviews and focus groups with clinicians (4 GPs, 5 endocrinologists, and 3 diabetes educators) and 6 people with T2D. Following refinements, 8 GPs participated in mock consultations in which they had access to GlycASSIST. Six people with T2D viewed a similar mock consultation. Participants provided feedback on the functionality of GlycASSIST and its role in supporting shared decision making (SDM) and treatment intensification.

**Results:**

Clinicians and people with T2D believed that GlycASSIST could support SDM (although this was not always observed in the mock consultations) and individualized treatment intensification. They recommended that GlycASSIST includes less information while maintaining relevance and credibility and using graphs and colors to enhance visual appeal. Maintaining clinical autonomy was important to GPs, as they wanted the capacity to override GlycASSIST’s recommendations when appropriate. Clinicians requested easier screen navigation and greater prescribing guidance and capabilities.

**Conclusions:**

GlycASSIST was perceived to achieve its purpose of facilitating treatment intensification and was acceptable to people with T2D and GPs. The GlycASSIST prototype is being refined based on these findings to prepare for quantitative evaluation.

## Introduction

Type 2 diabetes (T2D) affects more than 420 million people worldwide [1]. In Australia, T2D affects 1.2 million people, amounting to more than Aus $6 (US $4.3) billion annually in direct and indirect health care costs [2]. Early achievement and maintenance of glycated hemoglobin (HbA_1c_) in an appropriate target range reduces downstream complications and all-cause mortality [3]. Although the general target is HbA_1c_ of ≤7% (53 mmol/mol), the Australian Diabetes Society and international guidelines suggest that targets need to be individualized based on several factors, including age, duration of diabetes, comorbidities, and risk of hypoglycemia [4-6].

In Australia, as in many countries, most clinical care of people with T2D is based on general practice or primary care [7]. Treatment intensification by general practitioners (GPs) can help people with diabetes achieve glycemic targets [8,9]. However, more than 40% of Australian adults with T2D have an HbA_1c_ level above the target range [10]. Treatment intensification is typically delayed for 8 to 10 years, while HbA_1c_ remains out of target [11]. Barriers to GPs delivering evidence-based treatment intensification include lack of familiarity with clinical diabetes guidelines and the complex and rapidly changing treatment options for optimizing blood glucose levels (BGLs) [12-14]. Health system factors (eg, competing priorities in busy, reactive primary care settings [15]) and patient-related factors (eg, psychological resistance to insulin initiation [16]) can also play a role.

Clinical decision support (CDS) tools used in real time can enable GPs and people with diabetes to navigate this complex environment and intensify treatment in an appropriate and timely manner to achieve personalized HbA_1c_ targets. CDS tools and evidence-based electronic support can improve the process of care measures [17-19], the use of guidelines by GPs [20,21], and outcomes such as HbA_1c_ [22-25], without substantially increasing health care expenditure [26]. They can also reduce consultation duration and increase screening (eg, lipids) for complications associated with T2D [27,28].

Using CDS tools in real time during the consultation may also encourage treatment-specific conversations between GPs and people with diabetes, supporting shared decision making (SDM), an important aspect of quality care. A CDS tool embedded within an electronic medical record (EMR) has the added benefit of automatically extracting and using information present in the EMR to guide personalized clinical care. Until recently, most CDS tools have focused on helping people with diabetes achieve a standardized set of diabetes goals (eg, HbA_1c_ target of <7%) that are not necessarily individualized or person-centered [29]. Recently, CDS tools that encourage individualized diabetes care and are integrated within the EMR have become available [30,31]. There is a plethora of self-management apps for people with T2D, many with a focus on displaying blood glucose data with the option to add data about medications, diet, and exercise. Many of them can be shared with health professionals. However, at the point of clinical care, CDS tool design and efficacy are often inconsistent [20,32-34]. Few CDS tools combine the capacity for automatic deployment within the EMR in real time in the consultation with the capacity to make management recommendations (beyond specialized closed-loop insulin systems).

Utilization of CDS tools by health care professionals (HCPs) is low [32,35,36]. One way to increase uptake and utilization is through co-design that includes end users (eg, GPs and people with diabetes) [37,38]. The co-design theory suggests that technologies, services, and systems should be designed *with* the intended users, giving them the opportunity to inform development [39]. The focus is on engaging with end users to “jointly articulate ideas ... [and engage] ... with mock-ups and prototypes” [40].

The aim of this study was to use co-design principles to develop an EMR-based CDS tool for real-time use in consultations to support GPs and people with diabetes to select individualized HbA_1c_ targets and appropriate medication options together. In this paper, we describe the co-design cycles of feedback and tool refinement, drawing on first- and second-stage interviews and focus groups.

## Methods

The University of Melbourne Human Research Ethics Committee provided ethical approval for both stages (stage 1: 1851169 and stage 2: 1851535).

### Development of the GlycASSIST

**Figure 1:**
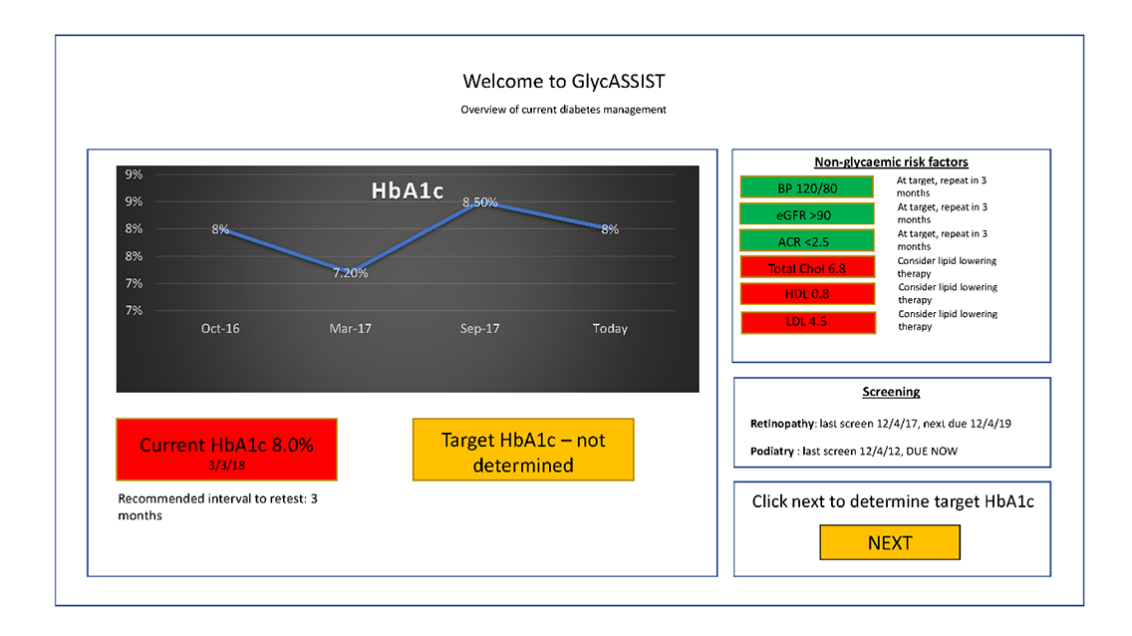
Initial prototype—page 1. ACR: albumin/creatinine ratio; BP: blood pressure; eGFR: estimated glomerular filtration rate; HbA1c: glycated hemoglobin; HDL: high-density lipoprotein; LDL: low-density lipoprotein; Total chol: total cholesterol.

We used an iterative two-stage co-design and refinement process to develop our CDS tool, GlycASSIST (Multimedia Appendix 1). This work was led by a multidisciplinary working group of academics and clinicians (with expertise in general practice, endocrinology, diabetes education, health psychology, behavioral science, and health informatics), who met monthly to critically review the design and refinement of GlycASSIST. A literature review identified T2D-related CDS tools currently available to GPs and summarized local guidelines [6,41] into evidence-based algorithms for personalizing HbA_1c_ targets (Multimedia Appendix 2), informing the design of the initial prototype before the first co-design stage (Figure 1). Algorithms also reflected cost to the patient via the Pharmaceutical Benefits Scheme (PBS). The PBS is a national system for providing medications at an affordable price to patients who meet specific prescribing criteria relating to current glycemic levels, previous medication use, intolerance, and contraindications to other medications. Given the number of medications available, it can be complex and time consuming for clinicians to navigate.

We used interviews and focus groups to engage clinicians and people with diabetes in the co-design process. Although each data collection method has particular strengths, we used both to accommodate timing and venue preferences for participants. Stage 1 and 2 participants were recruited through networks of the working group, advertisements on social media (eg, Twitter), and professional association electronic communications. GPs and people with diabetes participated in both stages. Endocrinologists and diabetes educators were engaged in stage 1. The study was conducted in 2018.

### Stage 1

Stage 1 interviews and focus groups were completed by trained facilitators (HM, ET, and BK) either in person or via web-based video chat. Clinicians were eligible to participate if they were a GP, an endocrinologist (with a case mix consisting of ≥50% people with T2D), or a diabetes educator. People with diabetes were eligible to participate in a separate focus group if they were aged 18 years or older, had T2D diagnosed for at least one year, and were taking glycemic medications. In-depth semistructured interviews and focus groups were designed, first, to identify the experiences and expectations of discussions about HbA_1c_ treatment targets and treatment decisions between clinicians and people with diabetes and, second, to gain feedback on the features and appearance of the initial GlycASSIST design (see Multimedia Appendices 3 and 4 for interview and focus group guides). Participants were shown the initial prototype (Figure 1 and Multimedia Appendix 5) as part of the interview and focus group and HCPs were also shown existing CDS tools [28,30,31]. The GlycASSIST prototype was further developed based on the feedback obtained.

### Stage 2

#### GP-Simulated Consultations and Interviews

Stage 2 involved simulated clinical consultations and think-aloud interviews to understand how GPs and people with diabetes interacted with the second prototype of GlycASSIST (Multimedia Appendices 6-11) and how it could be used to facilitate SDM and treatment intensification and to gain further feedback on the design. The prototype consisted of 2 main elements: an HbA_1c_ calculator to inform personalized HbA_1c_ targets and a medication intensification tool.

Stage 2 sessions with GPs were all facilitated by a GP (HM) and software programmer (SL) in person in a mock/simulation clinical environment. GPs were eligible to participate if they had not participated in stage 1. The GPs used a desktop computer setup with EMR software that directly interacted with GlycASSIST, allowing GlycASSIST to automatically extract information from the EMR and use it to make recommendations. For GP participants in stage 2, an *initial briefing* on the features of GlycASSIST was followed by testing in a *simulated consultation*, with a diabetes educator playing the part of a person with diabetes (a *simulated patient* named Maureen). The GP was asked to interact with the simulated patient as they would any person with diabetes seeking care. The simulated patient had basic knowledge of T2D management and played *Maureen*, using a script (Textbox 1) to respond to the anticipated questions asked by the GP and to improvise in response to unexpected questions. The simulated patient’s characteristics, contained within the script as well as additional clinical history, were uploaded to the EMR before the testing session to enable the GP to access clinical data as they might do routinely in a consultation and for GlycASSIST to extract and present Maureen’s information to the GP. Following the simulated consultation*, a semistructured interview* was conducted (see Multimedia Appendix 12 for the guide) to follow up on comments made during the simulation and to identify feedback specific to the use of GlycASSIST and its role in treatment intensification and SDM. Clinicians were encouraged to verbalize their thoughts, including barriers and facilitators using GlycASSIST and overall usability issues [42]. GPs were asked to explain to the facilitator why and how they interacted with GlycASSIST if they decided to use it.

**Summary of Maureen, a person with diabetes profiled in the clinical vignette used during the simulation of a clinical consultation in stage 2.**

- Maureen is a 55-year-old woman. She is seeing you/Dr Skari today to receive her most recent HbA_1c_ test results. She is reluctant to change her current medication plan but can be persuaded if the new medication is taken orally and is unlikely to result in weight gain. She does not want to check her blood sugar levels at home unless absolutely necessary
- Past medical history: No history of cardiovascular disease or stroke
- Social history: Lives alone. Carer for mother with dementia. Works part time as a receptionist. Her busy lifestyle can make it hard to manage her type 2 diabetes, but she reports that she takes her medication as prescribed
- Current medical history: type 2 diabetes for 5 years
- Current treatment: 2 g per day extended-release Metformin commenced 18 months ago. No complaints of dizziness or feeling faint
- Investigations: HbA_1c_ assessed 1 month ago=8%
- Lifestyle: Brisk walk 3 to 4 times per week for at least 30 min. Mediterranean diet but still occasionally has sweets
- Referrals: Currently seeing a dietitian. Last saw a podiatrist and an optometrist 3 months ago

#### Focus Groups With People With T2D

People with T2D were eligible to participate in 1 of the 2 *focus groups* based on the same criteria as stage 1 (some from stage 1 participated in stage 2). Each focus group (facilitated by HM and BK) involved participants *viewing a video of a simulated consultation* between a GP (HM) and *Maureen*, in which the GP used GlycASSIST with the person with diabetes to discuss an appropriate HbA_1c_ target and treatment plan. Participants were asked to comment on GlycASSIST and how it was used during the simulated consultation, including what they liked/disliked and suggest improvements (see Multimedia Appendix 13 for the guide).

### Data Analysis

All sessions were audio-recorded, transcribed verbatim, and checked for accuracy before importing to NVivo 10 for analysis (version 2012, QSR International Pty Ltd). Two investigators (BK and JF) completed an inductive thematic analysis to identify emerging themes across participant groups. Disagreements were resolved by consensus. Themes and illustrative quotes were reviewed by the entire study team.

## Results

### Stage 1

In total, 12 clinicians (4 GPs, 5 endocrinologists, and 3 diabetes educators) and 6 people with diabetes (3 males and 3 females; 4 people aged <65 years, and 4 people with diabetes duration >10 years) participated in stage 1 focus groups (range 96-112 min) or interviews (range 39-92 min). Six themes were identified around key characteristics participants valued in such a tool or would like to see changed or enhanced in the next iteration of the prototype (Table 1).

**Table 1:** Themes identified in stage 1 with exemplar quotes from health professionals and people with diabetes.

Themes	Health care professionals	People with diabetes^a^
Balancing information needs: relevance is key	“Too much information, and too much data entry, will just disengage people.” (GP1^b^) “I’ve got to go through it and I click things and it seems like that would take me longer...” (GP2) “Is there somewhere to prompt specialist referral?...Because, sometimes they’re referred years too late. Sometimes they’re referred years too early. It’s hard to strike a good balance. But, that may be relevant...” (Endocrinologist 2)	“You need more information, whether it’s linked to another page or something like that.” “Another thing they could add to the pop-up screen is asking ‘has a...plan been set up?’ because I find it so much easier.” “Its one case where I think information is not an information overload. [...]. I think information makes it acceptable and understandable.”
Credibility	“...whatever you do, you’ve got to be able to maintain this tool because it [the evidence] changes.” (Endocrinologist 1)	“I love hearing what is new around. I’m always interested in that.”
Using GlycASSIST to reduce prescribing complexity	“With all the changes [to the PBS schedule] I forget, off the top of my head, what you can and can’t do...” (GP1)	“...if you were having some of these side effects, the fact that there are other options is fabulous and the fact that they are listed up there [on GlycASSIST].”
The clinician, not GlycASSIST, retaining clinical autonomy	“...it would be nice if you could have this tool open, but still look at the rest of their EMR at the same time, rather than be locked out of it.” (GP1)	“I guess it all depends on how the doctor...explains it.”
Not just about medications	“I’d double down on her diet and exercise habits, which are not really provided here [on the GlycASSIST screen], particularly in the light of her concern about weight.” (Endocrinologist 3)	“...[GlycASSIST needs] more information on the lifestyle of that person...because to come up with this number [HbA_1c_ target] is very complex.”

^a^Focus group transcripts did not identify different individuals.

^b^GP: general practitioner.

#### Balancing Information Needs—Relevance Is Key

Clinicians wanted less information on the screen to avoid overcrowding and to reduce the number of clicks needed to progress to different GlycASSIST screens. Information retained on the screen needs to be relevant for T2D management. In contrast, people with diabetes wanted GlycASSIST to display a greater range of information (eg, weight, current medications, and comorbidities) to provide the GP with as much information as possible.

The presentation of all possible drug classes (n=8) was deemed important by clinicians and people with diabetes, but the inclusion of all side effects for each drug class was considered inappropriate by several clinicians, as some side effects are considered more common and/or serious than others. Instead, clinicians suggested listing only the most common and serious side effects. This was also supported by people with diabetes who felt it was important that medication information (both positive and negative) could be used by the GP during the discussion, where appropriate weighting could be given to each side effect.

Both clinicians and people with diabetes reported that prompts to establish a chronic disease management plan or refer to an appropriate specialist (eg, endocrinologist, dietician) would facilitate holistic and individualized care.

#### Credibility

Clinicians considered evidence-based recommendations critical to building trust in GlycASSIST. It was also important that the evidence was clear and updated as evidence changes. People with diabetes wanted updated information about new therapies.

#### Using GlycASSIST to Reduce Prescribing Complexity

Clinicians reported that GlycASSIST could be a useful tool to reduce the complexity associated with choosing diabetes glycemic medications for people with diabetes. Clinicians, especially GPs, indicated that GlycASSIST would be more useful if it included guidance on how to prescribe a medication that is subsidized by the Australian Government via the PBS. Having access to these complex subsidy regulations within GlycASSIST would make it easier to choose a medication affordable for the person with diabetes while also enabling the person with diabetes to choose a non–PBS-listed medication (ie, as a private nonsubsidized prescription) if they preferred. People with diabetes also saw the need for the tool to address complexity in the range of medication options available and in choosing based on side effects and cost.

#### The Clinician, Not GlycASSIST, Retaining Clinical Autonomy

It was important for GPs to make GlycASSIST work for them. For some, this meant having GlycASSIST directly generate a medication prescription, rather than only making recommendations. Others wanted GlycASSIST to perform time-saving tasks, such as entering clinical notes into the EMR, whereas for others, the autonomy to ignore or override HbA_1c_ targets or medications recommended by GlycASSIST was a priority. For people with diabetes, this was seen in how they wanted the tool to be something used by their doctor to personalize explanations and discussions, for example, in relation to medication contraindications or side effects. Several GPs indicated that it was important that GlycASSIST did not *pop up* on the screen, preferring a discrete icon that did not demand the GP’s attention and could be minimized if desired.

#### Not Just About Medications

Both clinicians and people with diabetes reported that including lifestyle modifications (eg, regular exercise) is important for holistic care of T2D. Therefore, several participants suggested having a lifestyle assessment prompt in GlycASSIST.

#### GlycASSIST Prototype Refinement

Not all the findings from stage 1 could be accommodated in the second iteration of the tool. Several features were maintained and strengthened in the refined second prototype (Multimedia Appendices 6-11). Evidence-based recommendations and associated algorithms were enhanced by adding more text demonstrating the latest evidence in *hover over* boxes and including an additional algorithm related to PBS prescribing rules. Formatting features, such as color coding and not having GlycASSIST *pop up* and consume too much space on the screen, were also maintained.

Enabling clinicians to retain autonomy in their use of the tool was maintained by ensuring clinicians could continue to override recommendations, select medication classes as they deemed appropriate (ie, whether PBS is listed or not), and modify the auto-populated fields. However, it was not possible for GlycASSIST to have the capability to populate the EMR with clinical notes or generate prescriptions (rather than returning manually to the EMR and prescribing from there). These features would be possible through collaboration with EMR software vendors, but this depth of integration was not possible in this pretesting study.

The main changes made to the GlycASSIST prototype focused on information presentation and trading broadness for specificity. All changes led to a more specific focus purely on glycemia and medications (Multimedia Appendices 6-11), with removal of extraneous information, such as reference to other cardiovascular disease (CVD) risk factors, referrals, and patient preferences. For example, questions that focused on the acceptability of weight gain and injectable treatments for people with diabetes and lifestyle concerns (eg, smoking history) were removed. Yes/no responses to these issues potentially oversimplified important topics for clinical discussion. Information about medication effects, side effects, and administration were retained within the medication summary boxes (drawing on local evidence-based resources [43] to facilitate discussion). Focusing only on glycemic medications also simplified the tool, which was valued by both clinicians and people with diabetes. Finally, some changes were made to enhance the visual appeal of the tool. Clinicians appreciated the use of color coding to indicate contrasting results and to draw their attention to important information. However, the size of the font used on the screen was too small in some places.

GlycASSIST was given the ability to display the availability of PBS cost-subsidy for each recommended medication class and their associated medications. In addition, a link to the Australian Government’s PBS webpage was provided [44].

### Stage 2

Eight GPs (4 females, 5 trained in Australia and 5 in practice for >10 years) participated in the stage 2 computer simulations (range 35-56 min, including consultations and follow-up interviews, but not including the initial briefing). Six people with diabetes (4 women, 3 aged >65 years and 2 with T2D >10 years) participated in 1 of the 2 focus groups (range 78-83 min). Four themes were identified during the simulated consultations with GPs, debriefing interviews with GPs, and focus groups with people with diabetes (Table 2).

**Table 2:** Themes identified in stage 2 and exemplar quotes from general practitioners and people with diabetes.

Themes	General practitioners	People with diabetes^a^
Using GlycASSIST to support SDM^b^	“This kind of decision making with patients, I'd normally say, ‘This is what I recommend and what do you think?’ I'd probably have this [SDM] conversation in my head.” (GP2^c^) “I thought it was quite handy actually, not just to me but I could talk through it with a patient.” (GP3, registrar) “...you can see all the medications and tick them off to say, ‘This is what we’ve got’. It wouldn’t be uncommon for those questions to come out, ‘Do we add it? Do I stop it? Why am I taking this? Side effects?’ It’s a good prompt to say, ‘Am I actually giving the right one here?’” (GP3)	“She had full access to what he was looking at too, because generally you can’t see the screen. The fact that they basically shared that screen [was good].” “...it’s almost too much information coming in. Its fine to have it all there in front of you but I just think...it’s a bit overwhelming.” “...It allowed the doctor to stop and pause...[...]. not assume.” “...it gave him a starting point and then he went through quite systematic steps..[...].. the patient was curious, and then going through all the options.”
GlycASSIST features	“...usually I’ll have to bring up diabetic guidelines and bring up therapeutic guidelines...Whereas, with the GlycASSIST, it was all there...I didn’t need to move up and about, so it actually shortened the consultation time.” (GP5)	“They [GP and the person with diabetes in video] talked at length in the consultation about the exercise she was doing. There’s nothing related to exercise [in GlycASSIST]...that’s part of the treatment.” “There’s a lot of people with diabetes who are on low income or pensions so it’s a really good thing to include that [PBS information].”
Visibility and information presentation	“...that was quite easy to use. That was very basic. It was clear and efficient, I could understand...and make a conscious decision. I thought that was good” (GP5) “If it wasn’t that easy to access...I’m unlikely to go to the desktop to find it.” (GP6)	“...that you’re able to document it all and particularly get a printout of it...” “..I don’t think comes naturally to a lot of doctors to share what’s on the screen...It makes it so much more easy to understand in the way its set out and everything. It’s the same information but it’s done in a different way...”
Workflow and navigation	“I’m not familiar with them, so I went, well, we’ll take that one [medication] out, we’ll take that one out and then just pick the next one...” (GP6) “If you could just minimize it, I think it would be simpler...Minimize, do it, pull it up. It would bring you back to the same place.” (GP3) “...So, this is the trouble with the software...you have to go back twice.” (GP1)	“... having a tool like this at the clinic I go to, there [are] probably 20 doctors […] they’ll just log into it and have it all there in front of them...”

^a^Focus group transcripts did not identify different individuals.

^b^SDM: shared decision making.

^c^GP: general practitioner.

#### Using GlycASSIST to Support SDM

Clinicians reported that the second prototype of GlycASSIST could help them intensify treatment in collaboration with people with diabetes. They appreciated the advice about PBS availability for each recommended medication class and option. This helped GPs “comply with the [PBS] regulation” [GP1] while helping people with diabetes by ensuring that prescribed medications were affordable to them. It saved time by avoiding the need to read through the information-dense material on the government PBS website or within the EMR prescribing software.

Clinicians and people with diabetes perceived the HbA_1c_ calculator as useful for prompting collaborative conversations around appropriate HbA_1c_ targets, although several GPs were surprised by the lower recommended HbA_1c_ target of 6.5% and overrode it, independently suggesting that 7% was a more appropriate target. One GP cited concerns about hypoglycemia and clinician autonomy when overriding the target.

Some GPs appreciated how several individualized drug class recommendations were made by GlycASSIST, providing people with diabetes with a choice. However, some GPs wanted specific recommendations about the single most appropriate class for the person with diabetes, rather than being presented with a variety of clinically appropriate possibilities.

Several GPs suggested that although GlycASSIST could facilitate SDM around medication choice, they would be less inclined to discuss all options if they had already decided what medication they would prescribe. Rarely did the GP ask the simulated patient if they wanted to consider changes to their current medication management before presenting options using GlycASSIST. Furthermore, GPs rarely discussed all recommended medication classes with the person. Instead, most chose 1 or 2 classes that they deemed appropriate and presented these to the person to choose the final option based on their own preferences.

Experienced GPs suggested that GlycASSIST would be more useful for less-experienced GPs (eg, GP registrars) when choosing appropriate medication options for people with diabetes, despite stating that they themselves are not always aware of new medications and can forget existing ones.

People with diabetes mentioned that they would like to see and discuss the GP’s screen as they used GlycASSIST, potentially facilitating SDM. However, in the computer simulation, several GPs did not turn their screen to show the recommendations or information provided by GlycASSIST to the simulated patient. Some GPs and people with diabetes reported that the information presented by GlycASSIST might overwhelm the person (eg, by using medical jargon), and 1 GP mentioned that it would be physically impossible for them to show their screen to the person because of their office layout. One GP suggested that SDM was not something that they routinely engaged in, preferring to make clinical decisions themselves, without involving the person with diabetes.

#### GlycASSIST Features

Overall, the second prototype of the tool was mostly well received. Clinicians often reported that GlycASSIST was easy to use, having the potential to shorten consultations and avoiding the need to seek out guidelines midconsultation. However, more features were requested.

Several GPs and people with diabetes suggested adding features to the screen presenting recommended medication classes. GPs liked the tabular display of medication classes, but they suggested that more information was needed, such as more detailed descriptions of each class (eg, listing both the generic and brand names for medications belonging to each class, their dose, frequency, and delivery mechanism). Some GPs requested side effects to be explicitly categorized into *rare* and *common*.

Clinicians often recommended to the simulated patient that they may benefit from seeing a diabetes educator and other allied health professionals as well as adopting and maintaining lifestyle changes. This suggests that using GlycASSIST did not dominate the consultation and still allowed the GP to address other important elements of T2D management. Some GPs requested additional features to further support them to deliver this care, for example, an integrated link to a *people with diabetes friendly* handout, including information appropriate for people with low health literacy. Several GPs suggested that GlycASSIST could present CVD risk factors (eg, lipid profile) and allow for CVD risk calculation. Finally, some GPs recommended having prompts to refer to allied health professionals. This was also suggested by people with diabetes as well as a prompt for the GP to talk with them about lifestyle modifications as part of their T2D management.

#### Visual Appeal and Information Presentation

Several GPs and people with diabetes reported that they liked how information was presented on the GlycASSIST screens. Most of the positive feedback was related to the graphical representation of HbA_1c_ results, enabling a trend to appear, and the tabular display of medication class choices. Overall, this layout made it easy to obtain and understand information. The use of color was also reported as appealing, with 1 GP and some people with diabetes also recommending the use of images, such as needles and syringes, when referring to injectable medications.

Clinicians and people with diabetes indicated that less information on the screen made it easier to interact with GlycASSIST. However, they were aware that a compromise must be met where relevant information remained accessible. Some GPs suggested that additional *hover over* boxes be used, enabling the GP to see important information (eg, updated evidence for suggestions and considerations for assessing the risk of hypoglycemia).

Some available information was not clear to some GPs. Some did not notice that the person’s current medications and estimated glomerular filtration rate (a measure of renal function, extracted from the pathology results section of the EMR) were presented on the screen, so they sought that information manually from the EMR. Clinicians and people with diabetes also reported that the text size needed to be larger.

One GP mentioned that the subtle but obvious appearance of the GlycASSIST icon in the corner of the desktop increased the likelihood that she would click on it, suggesting that easy access and less dominating nature of the tool may be valued.

The presentation of relevant medication information, such as expected HbA_1c_ reduction, weight loss, and side effects such as nausea and genitourinary infections, was deemed important for SDM by GPs and appeared to be reflective of recommendations made in clinical guidelines.

#### Workflow and Navigation

Most GPs began the simulated consultation by asking the simulated patient general questions (eg, “How’s work going?”), assessing lifestyle factors (eg, “Are you walking every day?”), and asking about their ability to manage their diabetes (eg, “Are you taking your tablets as prescribed?”) before opening GlycASSIST.

GlycASSIST was usually opened once the GP wanted to talk about the simulated patient’s HbA_1c_, although several GPs accessed HbA_1c_ data from the investigation panel in the EMR first and then used GlycASSIST to demonstrate the trend in results over time. Clinicians progressed smoothly to the screen presenting the recommended medication classes once they had suggested to the person that their HbA_1c_ could be lowered. Once on this screen, some GPs discussed the classes with which they were most familiar and disregarded those that were unfamiliar.

Some clinicians progressed from the screen presenting the recommended medication classes to choose a specific medication to prescribe, but others found it difficult to identify a tab to facilitate this. Occasionally, GPs wanted to leave GlycASSIST to use the EMR (eg, to calculate CVD risk) or go back to the previous screen. Most GPs found these switching tasks difficult.

## Discussion

### Principal Findings

The aim of this study was to co-design and refine a prototype EMR-based CDS tool (*GlycASSIST*) for real time, in consultation use to support treatment intensification and SDM between GPs and people with diabetes when choosing HbA_1c_ targets and diabetes medications. The co-design process supported an iterative refinement of the tool based on early user experience. The findings from stage 1 interviews and focus groups with clinicians and people with diabetes indicated that GlycASSIST was perceived as useful for T2D management but required modifications to improve information clarity and functionality. Modifications were made to reduce the amount of information on the screens and to add recommendations regarding PBS availability, making the information presented more specific to glycemic medications. Stage 2 findings suggested that these changes were well received by GPs and people with diabetes, but more changes were required. Participants perceived GlycASSIST as useful for SDM and presented well. However, the participants recommended additional features. GPs wanted more information describing medications, added functionality (eg, being able to prescribe medications using GlycASSIST), and better navigation (eg, being able to return easily to a previous screen). People with diabetes wanted additional prompts for discussion of lifestyle, suggesting that such a feature in the tool may help overcome barriers that people with diabetes have raised on these topics.

Unlike existing CDS tools, GlycASSIST is unique in its specific focus on individualization of both HbA_1c_ targets and medication recommendations, resulting in recommendations tailored to the individual and based on both clinical and personal factors. Few existing CDS tools are EMR-integrated [28,30], and none calculate both an individualized HbA_1c_ target *and* make individualized medication recommendations on evidence-based, person-centered factors. Integration with the EMR is the strength of GlycASSIST, as it provides enough information and drug-specific advice to facilitate a conversation between the GP and person with diabetes about the most appropriate medication for them.

GlycASSIST was considered to make treatment intensification easier for both experienced GPs and registrars. This was based on the functionality of GlycASSIST (eg, using evidence-based algorithms to inform the presentation of appropriate medication classes and prescribing rules [45]) and its content (eg, including all T2D medication classes). Both clinicians and people with diabetes also felt that GlycASSIST has the potential to facilitate SDM, prompting discussion around medication side effects and outcome preferences. However, GPs in our study often overrode the HbA_1c_ target suggested by GlycASSIST and primarily discussed medication classes with which they were most familiar. Our findings suggest that although GlycASSIST might provide helpful recommendations, these recommendations do not necessarily inform clinical decisions or collaborative shared approaches to treatment choices. In a study in the United States, although >60% of physicians opened the Diabetes Wizard during a randomized controlled trial (RCT), few were still using it 1 year after incentives (and the intervention period) ended, despite most physicians being satisfied with the tool [30]. Some clinicians found it challenging to make explicit and share the conversation about medication choice that they would usually have *in their head*. Furthermore, the use of GlycASSIST in practice did not automatically lead to engagement of people with diabetes in SDM. Simply having the information and tools available to GPs may not necessarily lead to SDM if this is not the usual consulting style of a given practitioner. It was beyond the remit of our small pretesting study to include specific training or intervention to enhance SDM. Although GlycASSIST has the potential to save time in consultations and aid clinical recommendations, additional behavior change strategies may be required to facilitate uptake and reduce clinical inertia when GlycASSIST is tested experimentally [46,47].

GlycASSIST was designed specifically for (and explicitly limited to) 2 tasks: assisting GPs and people with diabetes to choose individualized HbA_1c_ targets and T2D medications. However, this explicit focus exists in tension with the ideal of holistic and integrated diabetes care. Holistic care for people with T2D includes the important role that lifestyle interventions can play in achieving glycemic targets. It also involves linking T2D to the broader clinical issue of CVD. GlycASSIST could, for example, have the capability for automated calculation and display of individualized CVD risk and highlight modifiable risk factors and lifestyle interventions for CVD. It has been recommended that T2D CDS tools include all domains of T2D care (eg, smoking frequency and other CVD risk factors) to facilitate increased and ongoing use [36]. This may generate a practical *one-stop shop* for GPs to access multimorbidity CDS. Thus, a targeted and focused tool such as GlycASSIST may benefit from integration into a more comprehensive chronic disease management tool, protecting its specificity while being embedded within a holistic approach to care by partnering GlycASSIST with related chronic disease management tools.

The evidence-based nature of recommendations generated by GlycASSIST was mentioned frequently as beneficial and necessary to facilitate treatment intensification, assuming that GlycASSIST remains up-to-date. The features that allow GlycASSIST to make treatment intensification easier are time, funding, and resource intensive [45]. A tool such as GlycASSIST needs continual updating as new evidence and clinical guidelines become available. Further development and testing of GlycASSIST in an RCT to establish efficacy at improving patient outcomes, SDM, and overcoming treatment inertia is necessary. At this stage, no plans exist for commercialization.

### Strengths and Limitations

A strength of our study is that it is specific to the local context of the anticipated users (eg, by incorporating PBS rules and only medications approved for use in Australia) and provides person-centered individualized support for both establishing HbA_1c_ targets and choosing appropriate medications with people with diabetes, something not found in existing CDS tools specific to T2D management. With the multiplicity of EMR software providers in Australia and worldwide, integrated CDS tools must be developed locally to be clinically useful, incorporating their own prescribing rules. Co-design was used to enhance the design of GlycASSIST, ensuring that it met the needs of end users: both clinicians and people with diabetes. This process enabled the testing of GlycASSIST with end users to explore their interest in and interaction with GlycASSIST, which is critical for identifying both barriers to and enablers of adoption in real-world clinical settings [38,45].

One limitation, as discussed earlier, is that GlycASSIST focuses on HbA_1c_ only, although this was a compromise made explicitly and as part of our co-design process. Within that focus on glucose levels, GlycASSIST addressed pharmacological treatment intensification only, not lifestyle interventions. This limitation was a compromise, made consciously based on feedback about avoiding too much information in the tool. Another limitation of the tool is that it does not allow for uploading finger-prick BGL data. Many industry-based and other apps allow for the entry and display of individual BGL data, which could be used alongside GlycASSIST. Our tool focused on automated data extraction from the EMR for use in a consultation. Integration with the many BGL display apps could be an area for future development. Our tool did not include recommendations for deintensifying therapy. Again, this could be an area for future development. In this early formative study, GlycASSIST was developed to integrate with only 1 commercially available EMR software (Best Practice). There are multiple EMR vendors in Australia, and any future widespread implementation of GlycASSIST would require collaboration with the vendors. Our study was a formative evaluation and co-design of a prototype tool, so we did not test GlycASSIST in actual clinical practice. The GPs participating in the stage 2 computer simulations were provided with a comprehensive vignette of the simulated patient (*Maureen*). The details within the vignette might have inadvertently prompted the GP to make certain assessments, search for investigations, or make certain treatment suggestions (eg, based on CVD history). It is unclear if, and to what extent, this detail influenced the GPs’ interactions with GlycASSIST or their comments about it, especially regarding suitability for clinical practice. Furthermore, the presence of the researcher and the simulated nature of the pilot testing likely impacted the way the GP interacted with the tool. Finally, GPs indicated that they participated because they were interested in GlycASSIST and clearly saw a clinical need for the tool, suggesting that their views may not be representative of the broader clinician community.

### Conclusions

Our prototype has both face and content validity as well as acceptability and feasibility. Co-design incorporating local context and end user views led to a targeted tool that retained the capacity for integration with broader chronic disease management support. GlycASSIST requires additional refinement and evaluation, possibly as part of a suite of chronic disease management CDS tools, to establish its efficacy and broader acceptability for use in Australian general practice.

## Appendix

Multimedia Appendix 1 Codesign phases.

Multimedia Appendix 2 HbA1c algorithm.

Multimedia Appendix 3 Stage 1 Interview guide.

Multimedia Appendix 4 Stage 1 focus group guide.

Multimedia Appendix 5 Initial prototype treatment intensification page.

Multimedia Appendix 6 Second prototype - button within the EMR.

Multimedia Appendix 7 Second prototype - opening page.

Multimedia Appendix 8 Second prototype - treatment options page.

Multimedia Appendix 9 Second prototype - expanded treatment options.

Multimedia Appendix 10 Second prototype - prescribing page.

Multimedia Appendix 11 Prototype 2 additional screens 6.

Multimedia Appendix 12 Stage 2 GP Interview guide.

Multimedia Appendix 13 Stage 2 focus group guide.

## Abbreviations

ACBRD: Australian Centre for Behavioural Research in Diabetes
BGL: blood glucose level
CDS: clinical decision support
CVD: cardiovascular disease
EMR: electronic medical record
GP: general practitioner
HbA_1c_: glycated hemoglobin
HCP: health care professional
PBS: Pharmaceutical Benefits Scheme
RCT: randomized controlled trial
SDM: shared decision making
T2D: type 2 diabetes
