# Supplementary figures and images for: Guiding Glucose Management Discussions Among Adults With Type 2 Diabetes in General Practice: Development and Pretesting of a Clinical Decision Support Tool Prototype Embedded in an Electronic Medical Record
Source: JMIR Form Res. 2020 Sep 2;4(9):e17785. doi: 10.2196/17785 (PMC7495264; doi:10.2196/17785)

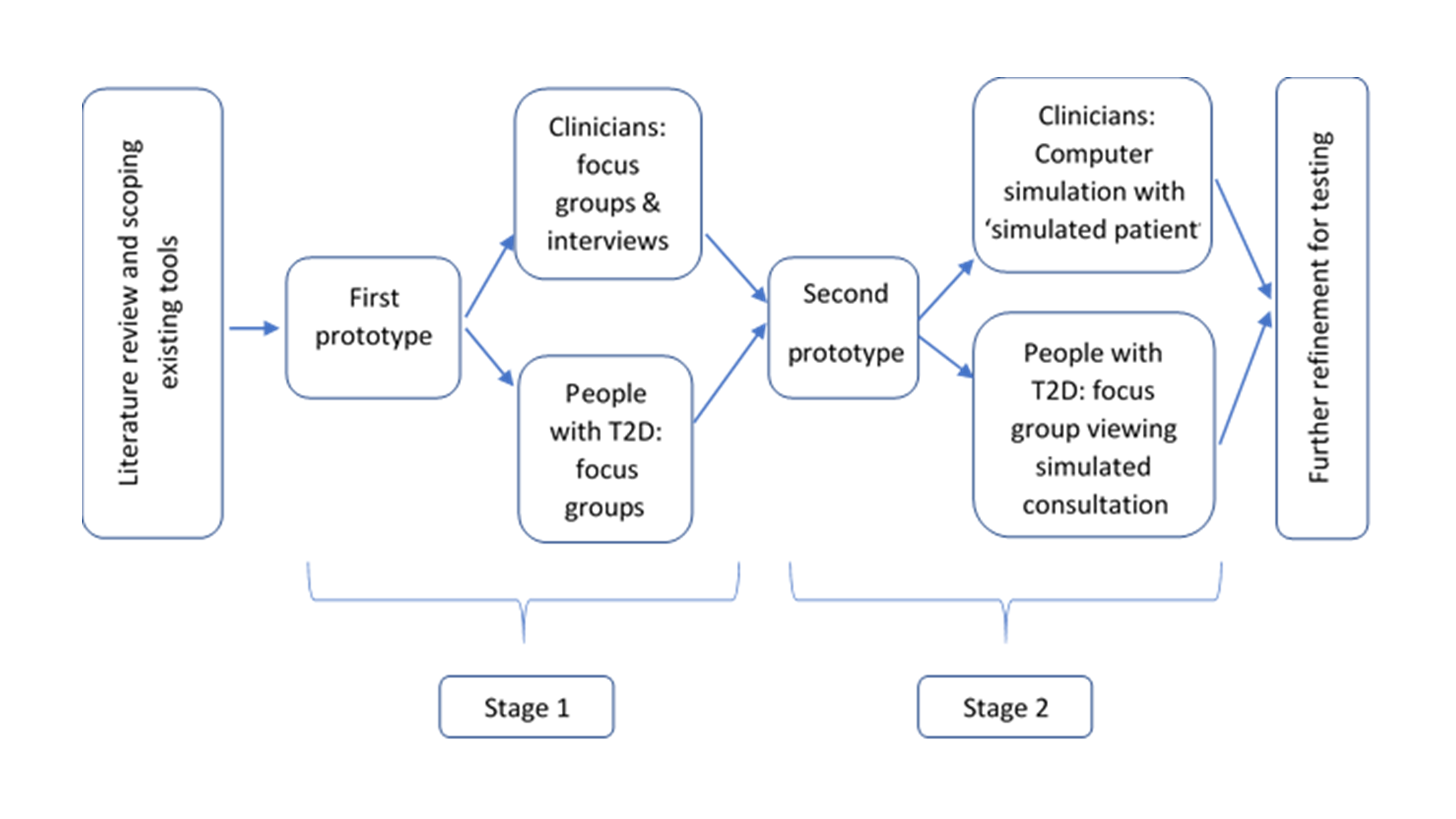

Supplement: Multimedia Appendix 1 [file formative_v4i9e17785_app1.png]

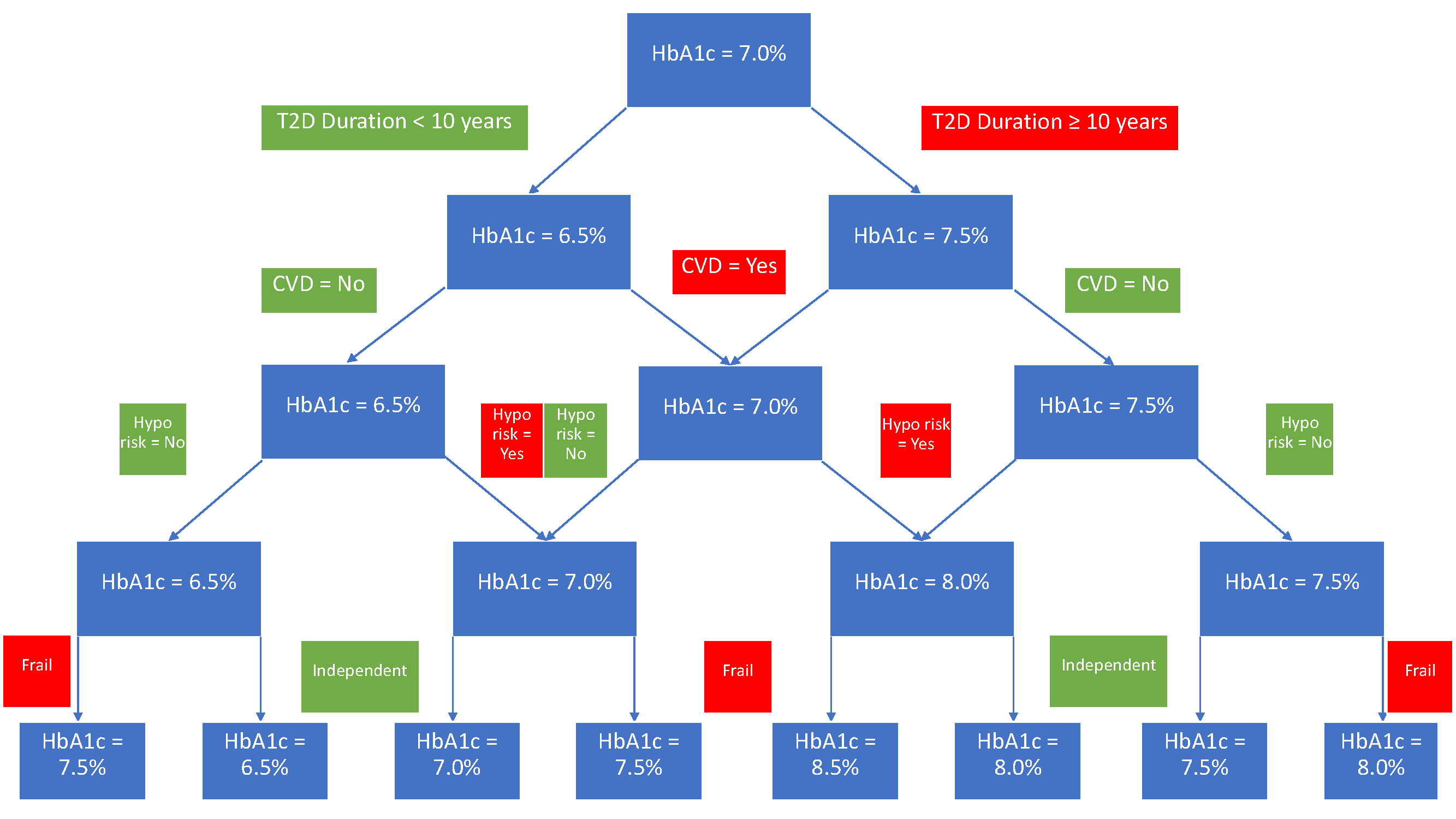

Supplement: Multimedia Appendix 2 [file formative_v4i9e17785_app2.png]

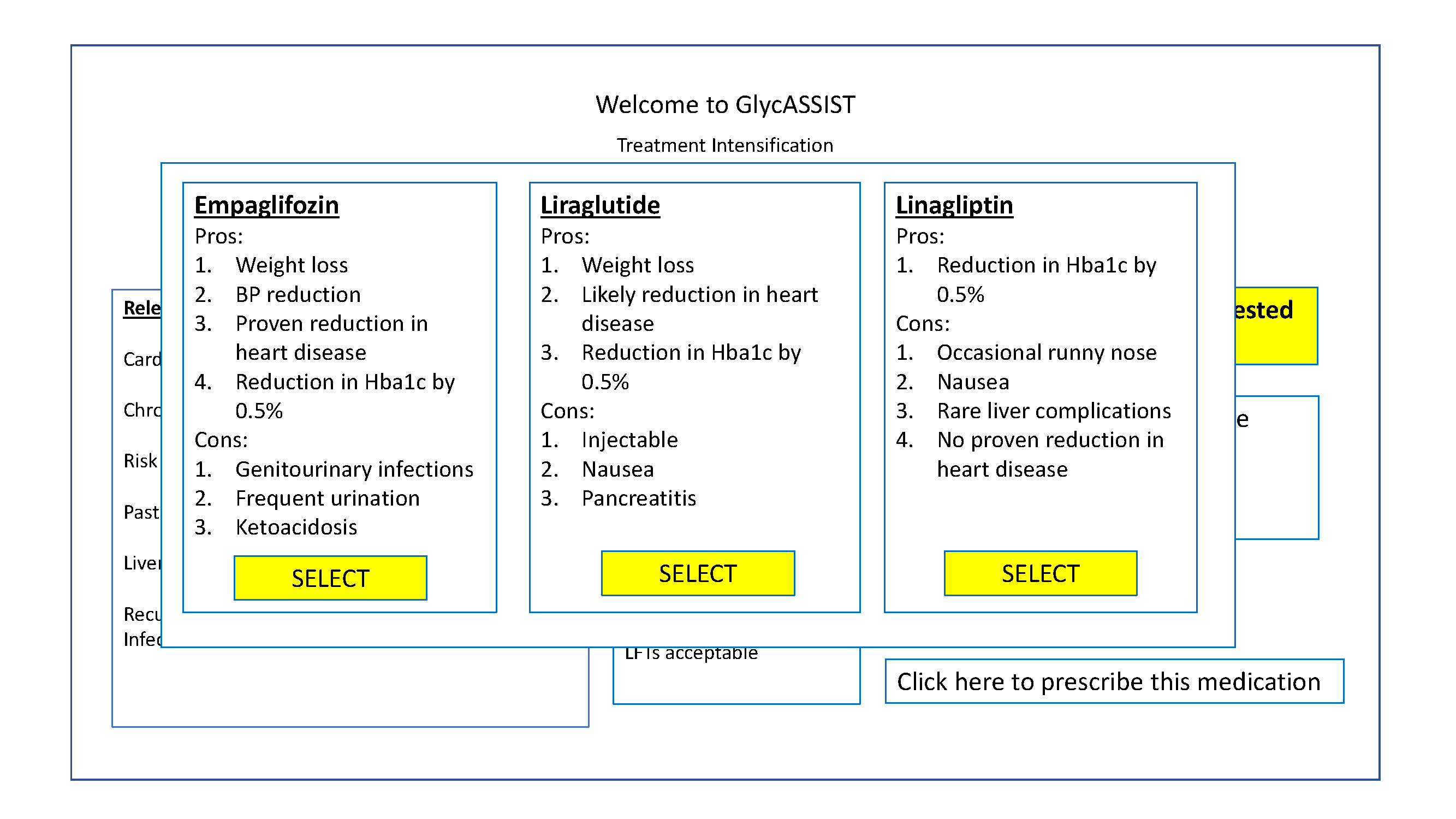

Supplement: Multimedia Appendix 5 [file formative_v4i9e17785_app5.png]

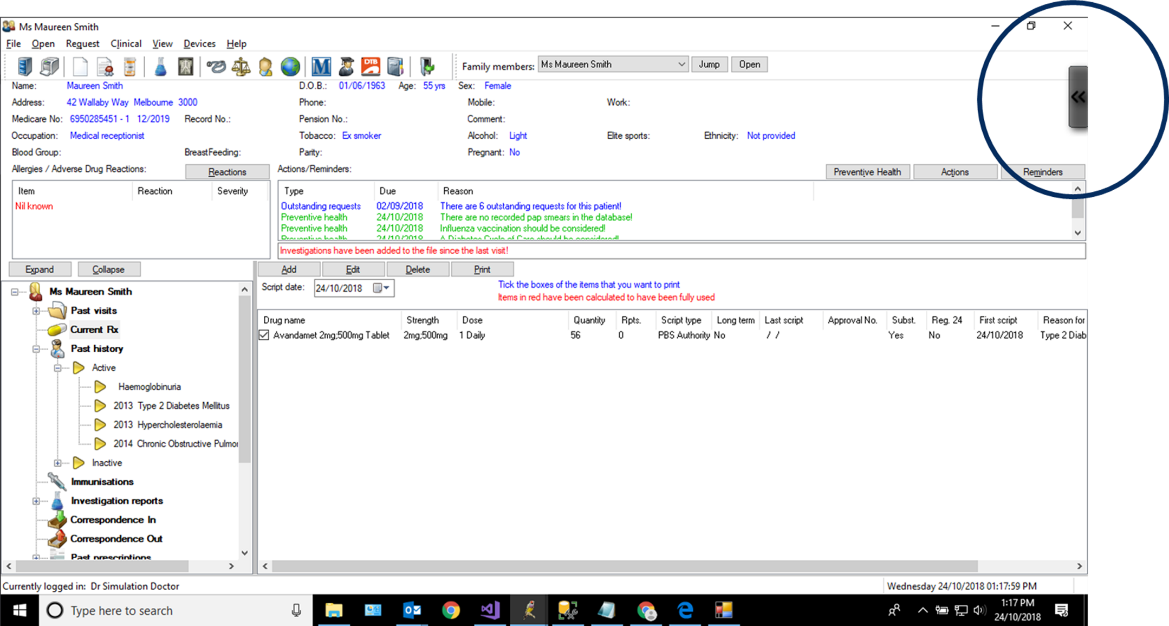

Supplement: Multimedia Appendix 6 [file formative_v4i9e17785_app6.png]

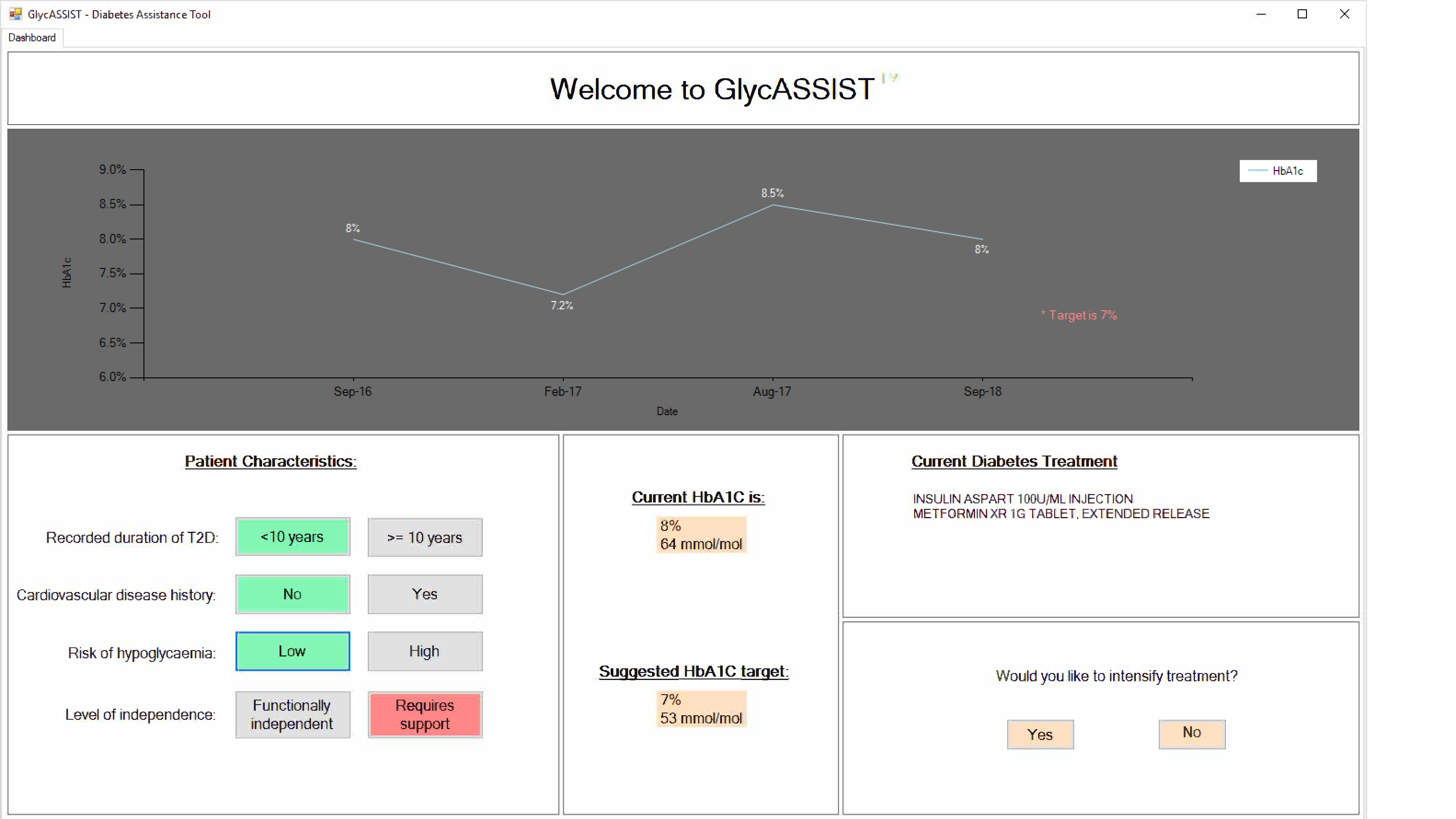

Supplement: Multimedia Appendix 7 [file formative_v4i9e17785_app7.png]

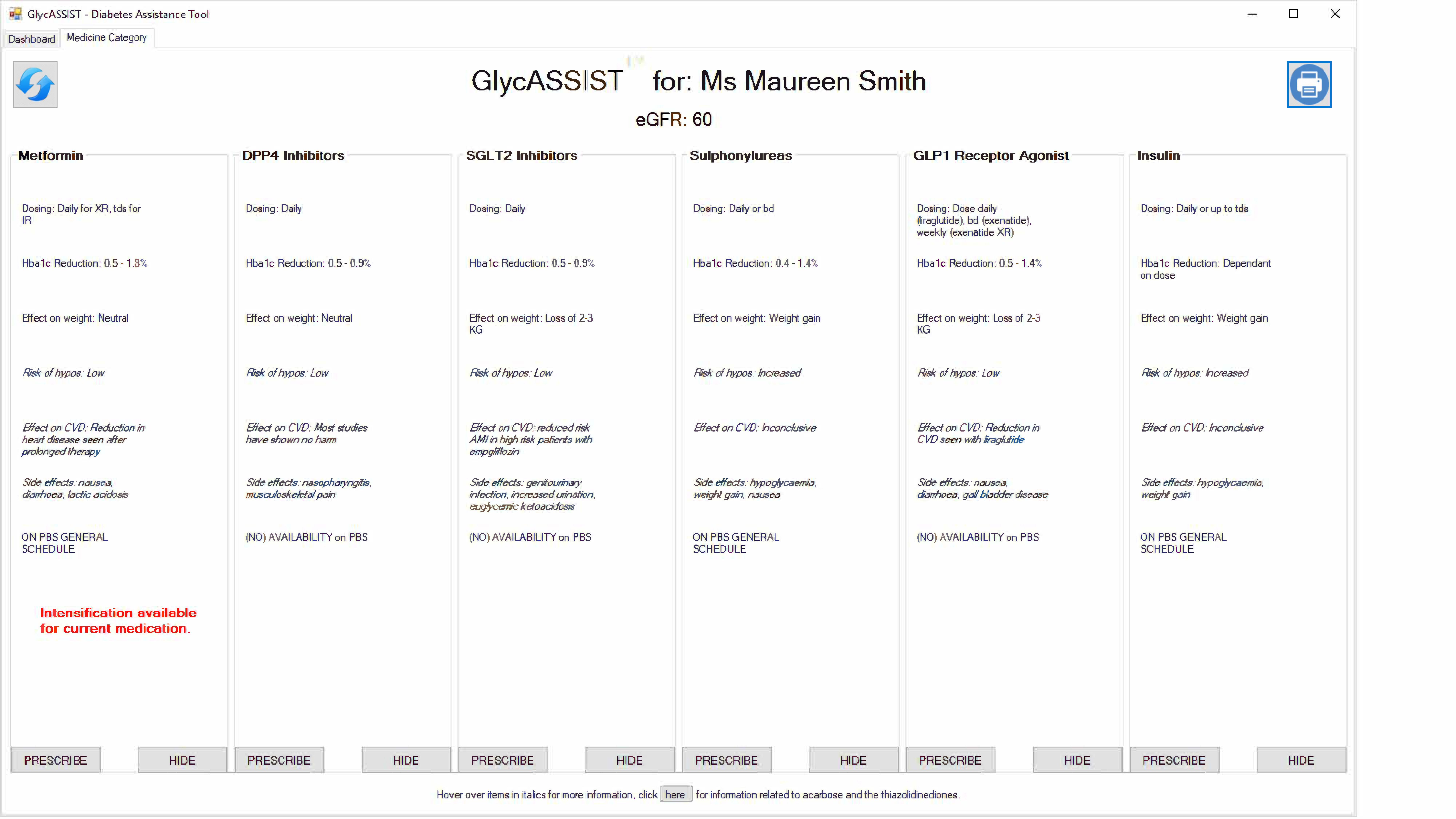

Supplement: Multimedia Appendix 8 [file formative_v4i9e17785_app8.png]

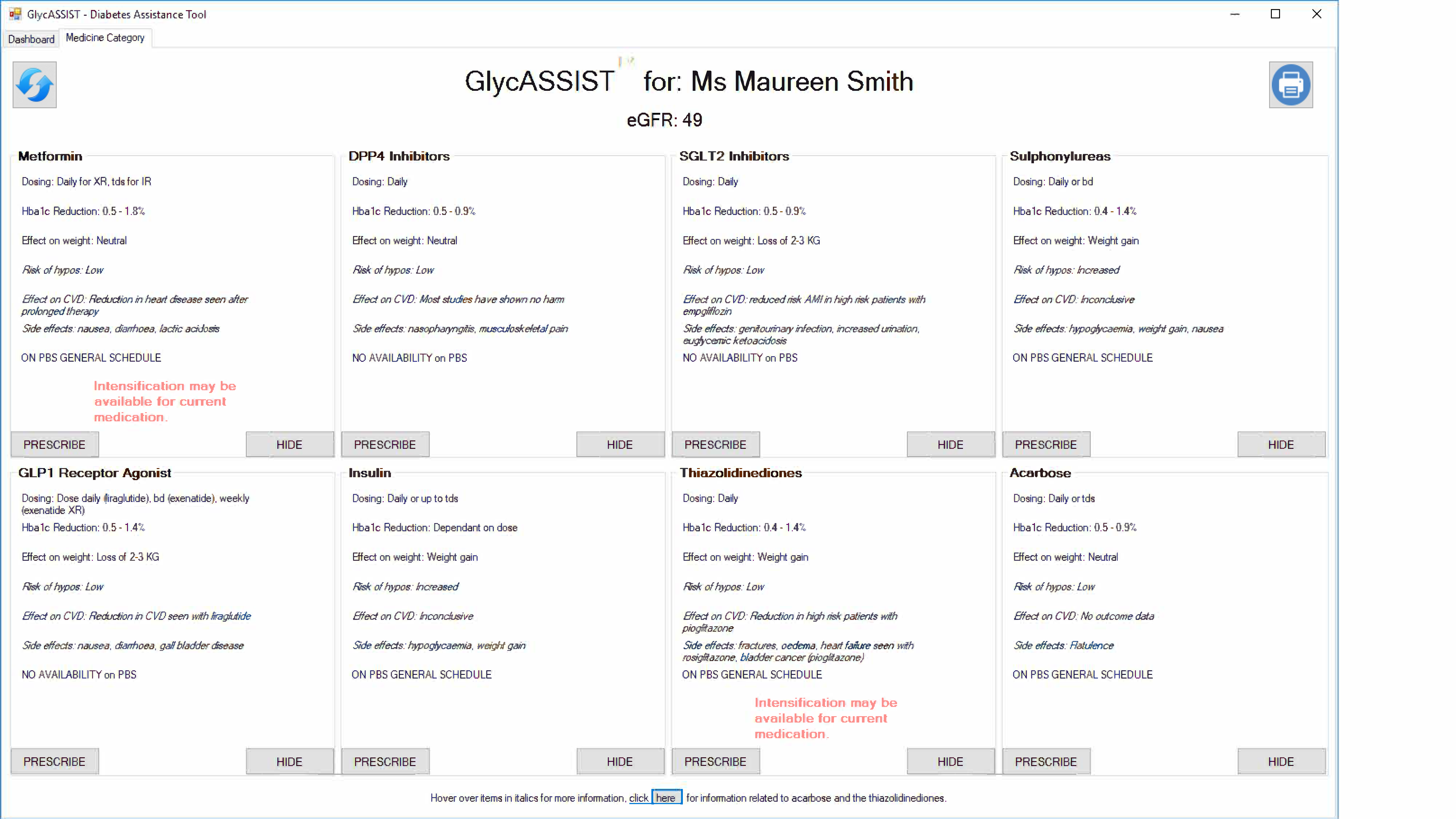

Supplement: Multimedia Appendix 9 [file formative_v4i9e17785_app9.png]

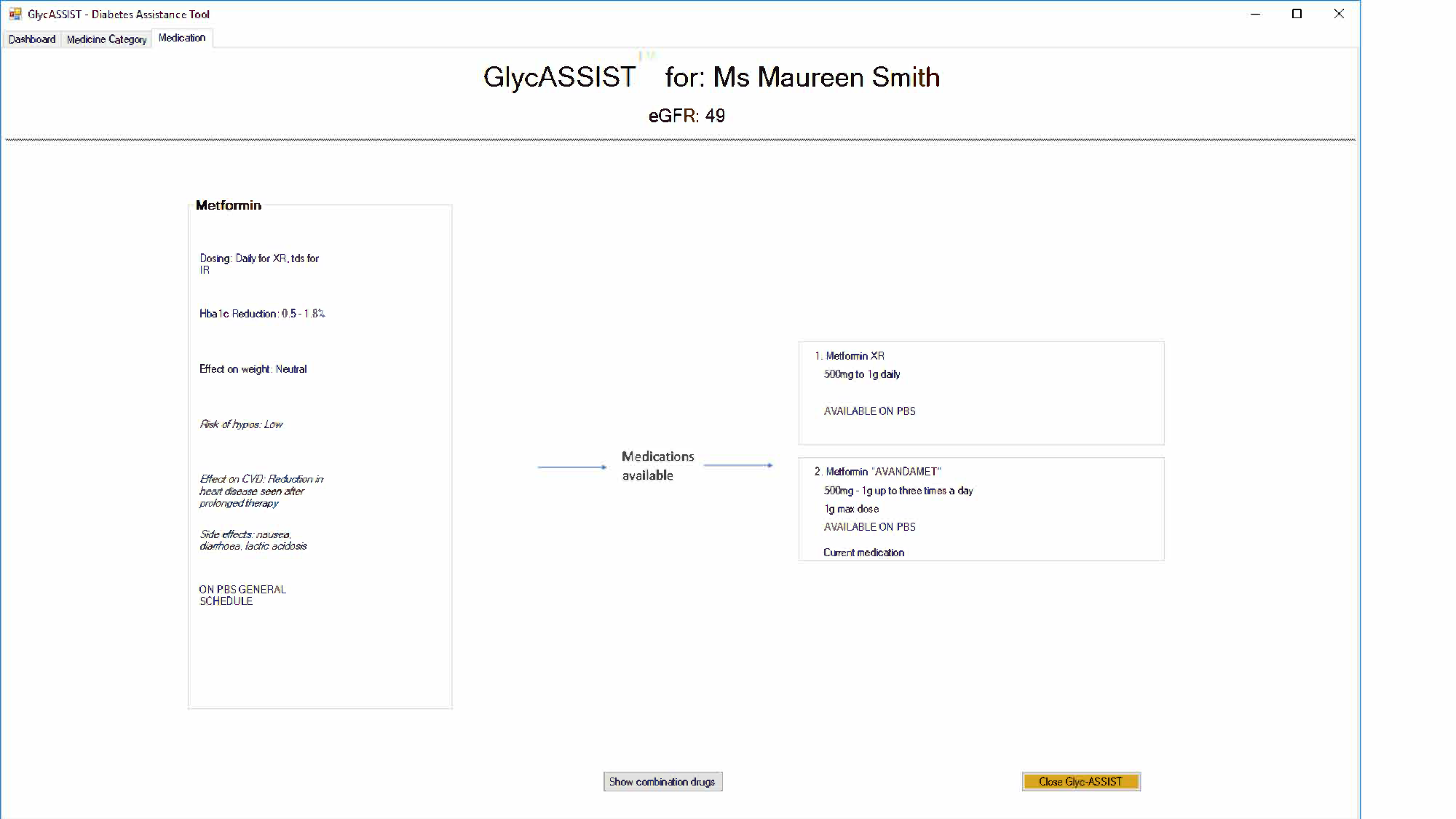

Supplement: Multimedia Appendix 10 [file formative_v4i9e17785_app10.png]

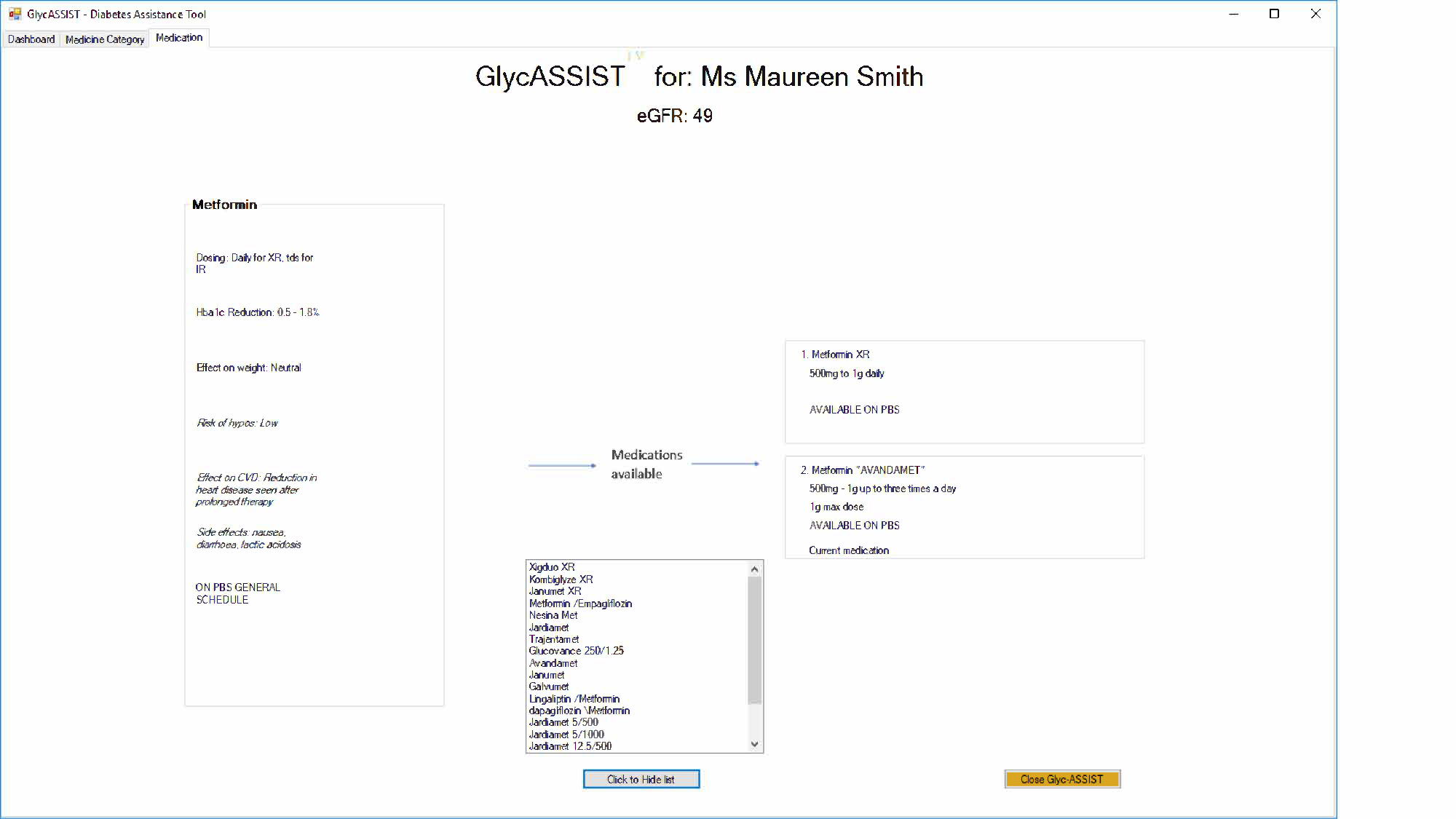

Supplement: Multimedia Appendix 11 [file formative_v4i9e17785_app11.png]
