# Supplementary material for: Guiding Glucose Management Discussions Among Adults With Type 2 Diabetes in General Practice: Development and Pretesting of a Clinical Decision Support Tool Prototype Embedded in an Electronic Medical Record
Source: JMIR Form Res. 2020 Sep 2;4(9):e17785. doi: 10.2196/17785 (PMC7495264; doi:10.2196/17785)
Supplement: Multimedia Appendix 3 [file formative_v4i9e17785_app3.pdf]

Clinician Guide: Stage 1

Script

Prompt: The idea is we will use the information gathered today, as well as the current evidence and guidelines, to make a clinical decision support tool that would be based in the medical records (such as medical director and best practice).

This program would help you and the patient engage in shared decision making to decide on a HbA1c target for that patient, and then suggest medications to use.

Once a target has been chosen, the program would then generate graphical or picture-based data to help you and the patient engage in shared decision making

1. What are your initial thoughts?
2. Is this something that would be useful in your practice?
3. Have you used similar programs, perhaps with other chronic health conditions?

Prompt 1: A few similar tools already exist, I'll show you some pictures of what they look like, I'd like to hear your initial impressions.

This is diabetes wizard, a US developed tool which is based in the electronic medical records. It uses algorithms based on evidence and best practice guidelines to:

a. Suggest specific changes in medications for patients not at individualized hemoglobin A1c, blood pressure, or lipid targets
b. Suggest changes in treatment for patients with contraindications to existing treatments (eg, metformin use in renal insufficiency or congestive heart failure), or being treated with potentially risky drug combinations (eg, concomitant β-blocker and nondihydropyridine calcium channel blocker);
c. Suggest obtaining overdue laboratory tests, such as for potassium, serum creatinine, creatinine kinase, or liver function tests
d. Suggest short follow-up intervals, such as monthly visits, for patients not at goal, because more frequent visits are associated with better chronic disease out-comes in many clinical trials.

Questions:

1. What are your thoughts?
2. Is this easy for you to read?
3. Would you like anything else to be included?

Prompt 2. This is Diabetes Dashboard that automatically retrieves pathology data from the EMR and uses colours to let you see what screening the patient has had, or is now due for.

Green is good control, yellow is sub-optimal control, red is poor control. The result is asterisked if the recommended testing interval has been exceeded.

Questions:

1. What are your thoughts?
2. Is this easy for you to read?

3. Would you like anything else to be included?

Prompt 3: This is diascope, which allows you to input some data regarding the patient (for example, current medication, risks of hypos), it then gives you the average recommendation of a panel of diabetologists regarding different medication options.

Questions:

1. What are your thoughts?
2. Is this easy for you to read?
3. Would you like anything else to be included?
