# Supplementary material for: Guiding Glucose Management Discussions Among Adults With Type 2 Diabetes in General Practice: Development and Pretesting of a Clinical Decision Support Tool Prototype Embedded in an Electronic Medical Record
Source: JMIR Form Res. 2020 Sep 2;4(9):e17785. doi: 10.2196/17785 (PMC7495264; doi:10.2196/17785)
Supplement: Multimedia Appendix 4 [file formative_v4i9e17785_app4.pdf]

People with diabetes guide: Stage 1

Discussion topics/questions

Interviewer: We first want discuss your experience of managing your blood glucose. Other words for blood glucose are: blood sugars, sugar levels or maybe you say "HbA1c". All of these terms are used to refer to about the amount of glucose in your blood which is higher for people who have diabetes.

Questions (1,2,3) and prompts (a,b,c):

1. Would someone like to begin by sharing how they manage their blood glucose?
    a. How do you manage your diabetes (e.g. discussion of lifestyle and medications)
2. Do you have a blood glucose goal or target in mind?
    a. How do you feel about your target?
    b. How did you decide on your target?
3. How do you feel about your current blood glucose levels?
    a. Are they important/not important, why?
    b. Are you satisfied/not satisfied, why?
    c. What helps you to, or stands in the way, achieve or get closer to your target glucose level?

Interviewer: We now want to focus on your experiences of discussing diabetes with your health professional.

1. Does anyone see a health professional other than a GP for their diabetes check-ups?
2. Can you tell us about a usual diabetes check-up with your GP/Endo/health professional – what does it look like?
    a. What is your experience discussing your T2D management with you GP?
    d. What topics do you talk about with him/her?
    e. What information about yourself, your life and your diabetes management do you share with your GP? Are there other things you would like to share or be asked about?
3. Do you discuss your blood glucose or HbA1c targets with your GP/Endo?
    a. Can you tell us about this experience?
4. You are all using medications to manage your diabetes. Could anyone share with the group about their experience of the decision-making process to start medications?
    a. Why was this decision made?
    b. Who made the decision?
    c. Were you happy this the decision-making process?
5. What do you want to achieve by appropriately controlling your diabetes? What would you like to happen?
6. When discussing treatment options with your GP, is there anything you would like your GP to ask you about? Or is there anything you would like to like to be able to tell them?
    a. When discussing the best possible treatment options for you, how would you feel if you GP asked you about:
        i. Your social situation, such as whether you have enough social support or your finances
        ii. Your concerns about side-effects of treatment, such as hypoglycaemia
        iii. Your health literacy and confidence, such as your ability to understand and administer the medications discussed
    b. Are there any barriers to discussing these topics, or any other topics with your GP?

Interviewer: We now want to turn your attention to a tool we have designed. This tool is called GlycASSIST, which has the purpose of helping you and your health care professional to choose appropriate HbA1c targets and T2D medications with you. I will now take you though this tool and ask you what you think.

1. Overall, what do you think about this tool?
    a. Helpful?
    b. Clear?
    c. Relevant?
2. What do you like?
3. What do you not like?
4. What should be added?
5. What should be taken out?
