# Supplementary material for: Guiding Glucose Management Discussions Among Adults With Type 2 Diabetes in General Practice: Development and Pretesting of a Clinical Decision Support Tool Prototype Embedded in an Electronic Medical Record
Source: JMIR Form Res. 2020 Sep 2;4(9):e17785. doi: 10.2196/17785 (PMC7495264; doi:10.2196/17785)
Supplement: Multimedia Appendix 12 [file formative_v4i9e17785_app12.pdf]

<u>Clinician guide: Stage 2</u>

Welcome and thanks for participating in our study. My name is … and I am a …, I will be coordinating the interview today. The purpose of today's think-aloud interview is to help explore your thoughts and decision-making processes when managing patient with type 2 diabetes using a new clinical decision support tool, GlycASSIST. This will be done by having you use GlycASSIST on the computer to help you to intensify treatment for a pretend patient, Maureen.

I will demonstrate GlycASSIST to you anf let you play with it, but I ask that you voice your thoughts as you do so. Tell me exactly what you are thinking as you use GlycASSIST. After this, a near-live phase using a simulated patient will occur. I will ask you to treat the patient like you would in your usual clinical practice. However, please try to use GlycASSIST to support your decision making. I will ask you some questions about your experience using GlycASSIST afterwards.

The process should take about 60-90 minutes. I will be recording the audio from this interview so it can be transcribed. I will also be video recording the interview so we can look back and see if and how you used GlycASSIST to support your decision making. The data will be stored securely at the University of Melbourne in accordance with our data storage procedures.

There are no strictly right or wrong thoughts! This session is not an exam; we are not interested in how well you work with people to manage their T2D. We are simply interested in how, and if, you interact with GlycASSIST.

Ask the participant to start using GlycASSIST with the pretend case (Maureen) and encourage them to voice their thoughts.

Topic for discussion (if these questions have not been answered in the think-aloud process)

Ask the following questions

1. What are your thoughts?
    a. Did GlycASSIST help you during the consultation?
        i. If no, why not?
        ii. If yes, how?
2. Was the near-live consultation realistic?
    a. What was different/the same?
3. I noticed that you used/did not use GlycASSIST. Can you please tell me why you decided to use it or why you did not use it?
4. In what circumstances would you use GlycASSIST?
    a. What types of patients would you use this tool with?
5. What additional information do you think needs to be asked by the tool?
6. Do you trust the information provided by GlycASSIST?
    a. Does it 'fit' with what you know clinically?
7. What information do you think is being used to calculate the suggested HbA1c? Do you think any other information should be considered?
8. What functions do you like?
9. Should GlycASSIST have more functions?
10. If you would change anything, what would you change about GlycASSIST? What would you keep the same?
11. Is this tool useful?
12. Is it easy to use?

13. Would you use it clinically? How likely are you to use this clinically?
    a. Does GlycASSIST fit in your clinical workflow?
    b. Will you act on GlycASSIST's suggestions?
        i. If yes, why? All the time?
        ii. If no, why not? All the time?
14. Will GlycASSIST improve clinical management of the people with T2D that you see?
15. Is it intuitive?
16. Would you order the drug classes differently?
17. What barriers do you foresee to using GlycASSIST regularly (e.g. with all people with T2D)
18. What facilitators do you foresee to using GlycASSIST regularly (e.g. with all people with T2D)
19. Would you show your screen to your patient?

Participants will be asked questions from the following scales if time permits:

USE THE FOLLOWING SURVEY RESULTS TO PROMPT FURTHER DISCUSSION. WHY DID THEY SCORE THE ITEMS THE WAY THEY DID?

The PSSUQ Survey

The Post-Study Usability Questionnaire	Strongly Agree		Strongly Disagree	

		1	2	3	4	5	6	7		NA
1	Overall I am satisfied with how easy it is to use this system.	○	○	○	○	○	○	○		○
2	It was simple to use this system	○	○	○	○	○	○	○		
3	I was able to complete the tasks and scenarios quickly using this system.	○	○	○	○	○	○	○		
4	I felt comfortable using this system.	○	○	○	○	○	○	○		
5	It was easy to learn to use this system.	○	○	○	○	○	○	○		
6	I believe I could become productive quickly using this system.	○	○	○	○	○	○	○		
7	The system gave error messages that clearly told me how to fix problems.	○	○	○	○	○	○	○		
8	Whenever I made a mistake using the system, I could recover easily and quickly.	○	○	○	○	○	○	○		
9	The information (such as online help, on-screen messages and other documentation) provided with system was clear.	○	○	○	○	○	○	○		
10	It was easy for me to find the information I needed.	○	○	○	○	○	○	○		
11	The information was effective in helping me complete the tasks and scenarios.	○	○	○	○	○	○	○		
12	The organization of information on the system screens was clear.	○	○	○	○	○	○	○		
13	The interface* of this system was pleasant.	○	○	○	○	○	○	○		
14	I liked using the interface of this system.	○	○	○	○	○	○	○		
15	This system has all the functions and capabilities I expect it to have.	○	○	○	○	○	○	○		
16	Overall, I am satisfied with this system.	○	○	○	○	○	○	○		

The “interface” includes those items that you use to interact with the system. For example, some components of the interface are the keyboard, the mouse, the microphone, and the screens (including their graphics and language).

System Usability Scale

© Digital Equipment Corporation, 1986.

	Strongly disagree 1	2	3	4	Strongly agree 5
1. I think that I would like to use this system frequently					
2. I found the system unnecessarily complex					
3. I thought the system was easy to use					
4. I think that I would need the support of a technical person to be able to use this system					
5. I found the various functions in this system were well integrated					
6. I thought there was too much inconsistency in this system					
7. I would imagine that most people would learn to use this system very quickly					
8. I found the system very cumbersome to use					
9. I felt very confident using the system					
10. I needed to learn a lot of things before I could get going with this system					

Example Case Vignette: Maureen

Overview:

Maureen Smith is a 55-year-old female with a 5-year history of type 2 diabetes mellitus originally treated with lifestyle measures. Treatment was intensified with metformin 18 months ago. Despite this Maureen's HbA1c remains elevated at 8.0%

Past history:

COPD

Hypercholesterolaemia

Allergies:

NKDA

Medications:

Metformin 1g XR BD

Atorvastatin 10 mg daily

Ventolin inhaler PRN

Social History:

Works part-time as a medical receptionist

Carer for her elderly mother whom as Alzheimer's Dementia

Ex-smoker

Examination:

BP: 145/95

Height 170cm

Weight: 84 kg

BMI: 29.1

Waist circumference: 94cm

Retinopathy: nil

Signs of peripheral neuropathy: nil

Investigations and additional exam findings:

	October 2016	March 2017	Sept 2017	Today

Hba1c	8%	7.2%	8.5%	8%
eGFR	>90	>90	>90	>90
ACR	<3.5	<3.5	<3.5	<3.5
BP	140/85	138/83	148/85	145/95
Weight	80	82	82	84
BMI	27.7	28.4	28.4	29.1
Total Chol	4.5			
LDL	3.5			
HDL	1			

<u>Hba1c Trend:</u>

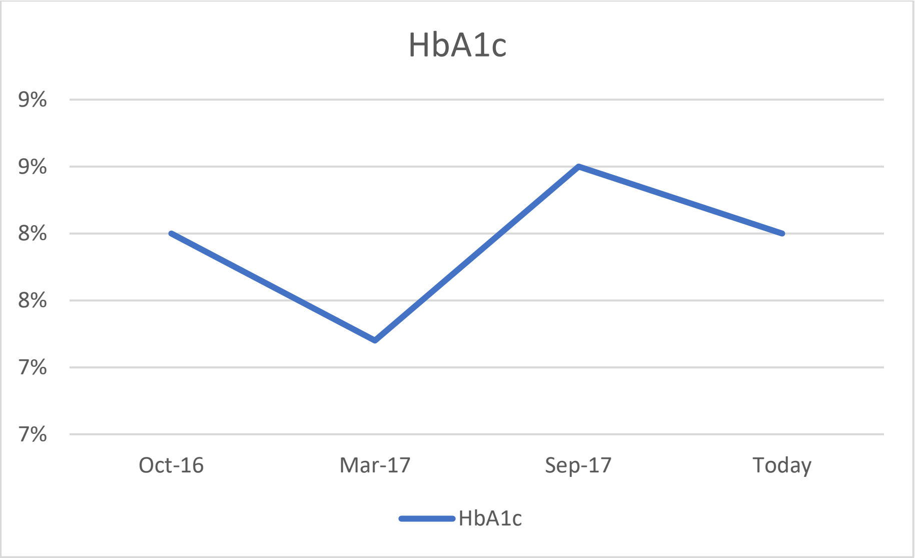

<u>Patient Preferences:</u>

Maureen's weight increased after her GP successfully counselled her regarding her smoking cessation. She was therefore frustrated when she was diagnosed with T2diabetes 1 year later. Despite referrals, Maureen is yet to see the optometrist and visit the diabetes nurse educator.

Maureen reluctantly commenced metformin, and again is hesitant regarding treatment intensification. Maureen views her diabetes as "mild" and finds diabetes management difficult as it competes with her duties as a carer.

She is also keen to avoid any weight gain, doesn't mind using an injectable agent, but would prefer not to have to measure blood sugars regularly.
