# Supplementary material for: Guiding Glucose Management Discussions Among Adults With Type 2 Diabetes in General Practice: Development and Pretesting of a Clinical Decision Support Tool Prototype Embedded in an Electronic Medical Record
Source: JMIR Form Res. 2020 Sep 2;4(9):e17785. doi: 10.2196/17785 (PMC7495264; doi:10.2196/17785)
Supplement: Multimedia Appendix 13 [file formative_v4i9e17785_app13.pdf]

# Person with T2D: Stage 2 script

Welcome and thanks for participating in our focus group discussion today. My name is … and I am a …, I will be coordinating the group discussion today. We also have… here today, who is…….

**1. Outline purpose of focus group**

You may remember from our last focus group that we are developing a new support tool called GlycASSIST. This tool is for GPs to use when seeing people with diabetes. It is designed to help the GP and person have a discussion and make decisions about diabetes management. Last time we met we discussed your experience of the decision-making processes when starting new diabetes medications and what information you think is important to consider. You also provided some initial feedback on a mock version of the tool we presented on PowerPoint slides. We have since gathered feedback from health professionals on the tool as well and developed a version of the tool which can be used in practice.

The purpose of today's focus group is to get your feedback on the more updated version of the tool and how it is being used in a GP setting. This will be done by having you view a video recording of the Glyc-ASSIST tool being used by one of the research team, a qualified GP in a pretend consultation with a person with type 2 diabetes. We are interested in any thoughts you have about the tool during the session. From your point of view, is the used of Glyc-ASSIT in the consultation, acceptable and useful. After watching the short 15 minute video we'll ask you some questions to help guide the discussion.

There are no strictly right or wrong thoughts! I expect there will be differing opinions and experiences which will be really useful for me to hear. Please respect each other's opinions and try not to interrupt if another person is talking.

**2. Topics for discussion (if the participant is struggling to say anything)**

Ask the following questions after viewing the video of the simulated consultation

1. First impressions: What are your thoughts?
    a. about the consultation/discussion
    b. about the use of the GlycASSIST tool
2. Was the consultation similar/different to your experience of diabetes consultations with your GP?
    a. How was it different/similar?
    b. What did you like/dislike about the consultation/discussion?
3. Do you think the use of the GlycASSIST tool was helpful in the discussion?
    a. How, for who, why?
4. What do you like about the GlycASSIST tool?
    a. What don't you like?
5. Would you like your GP to use this tool when helping you to make choices about how you manage your diabetes?
    a. Why/why not?
6. If you would change anything, what would you change about GlycASSIST?
    a. What would you keep the same?
    b. Is there anything else you would like included in the tool?
7. What problems might there be in using GlycASSIST in real consultations with people with type 2 diabetes?

Ethics approval number: **1851535**

8. Is there anything else you would like to add to the discussion?
    a. Do you have any other thoughts on the consultation or the GlycASSIST tool?
    b. bout the GlycASSIST tool and the consultation

Ethics approval number: **1851535**
